# Violence against Women and Stress-Related Disorders: Seeking for Associated Epigenetic Signatures, a Pilot Study

**DOI:** 10.3390/healthcare11020173

**Published:** 2023-01-06

**Authors:** Andrea Piccinini, Paolo Bailo, Giussy Barbara, Monica Miozzo, Silvia Tabano, Patrizia Colapietro, Claudia Farè, Silvia Maria Sirchia, Elena Battaglioli, Paola Bertuccio, Giulia Manenti, Laila Micci, Carlo La Vecchia, Alessandra Kustermann, Simona Gaudi

**Affiliations:** 1 Department of Biomedical Sciences for Health, Università degli Studi di Milano, 20100 Milan, Italy; 2 Service for Sexual and Domestic Violence( SVSeD), Fondazione IRCCS Ca’ Granda Ospedale Maggiore Policlinico, 20100 Milan, Italy; 3 Section of Legal Medicine, School of Law, University of Camerino, 62032 Camerino, Italy; 4 Gynecology Unit, Fondazione IRCCS Ca’ Granda Ospedale Maggiore Policlinico, 20100 Milan, Italy; 5 Department of Clinical Sciences and Community Health, Università degli Studi di Milano di Milano, 20100 Milan, Italy; 6 Medical Genetics, Department of Health Sciences, Università degli Studi di Milano, 20100 Milan, Italy; 7 Department of Pathophysiology and Transplantation, Università degli Studi di Milano, 20100 Milan, Italy; 8 Medical Genetics Unit, Fondazione IRCCS Ca’ Granda Ospedale Maggiore Policlinico, 20100 Milan, Italy; 9 Department of Medical Biotechnology and Translational Medicine, Università degli Studi di Milano, 20100 Milan, Italy; 10 Department of Public Health, Experimental and Forensic Medicine, University of Pavia, 27100 Pavia, Italy; 11 Department of Biotechnology and Biosciences, University of Milano-Bicocca, 20100 Milan, Italy; 12 Department of Environment and Health, Italian National Institute of Health, 00161 Rome, Italy

**Keywords:** sexual violence, intimate partner violence, epigenetics, PTSD, clinical forensic medicine, stress related disorders

## Abstract

Background: Violence against women is a relevant health and social problem with negative consequences on women’s health. The interaction between genome and environmental factors, such as violence, represents one of the major challenges in molecular medicine. The Epigenetics for WomEn (EpiWE) project is a multidisciplinary pilot study that intends to investigate the epigenetic signatures associated with intimate partner and sexual violence-induced stress-related disorders. Materials and Methods: In 2020, 62 women exposed to violence (13 women suffering from sexual violence and 49 from Intimate Partner Violence, IPV) and 50 women with no history of violence were recruited at the Service for Sexual and Domestic Violence. All women aged 18–65 were monitored for their physical and psychological conditions. Blood samples were collected, and DNAs were extracted and underwent the epigenetic analysis of 10 stress-related genes. Results: PTSD prevalence in victims was assessed at 8.1%. Quantitative methylation evaluation of the ten selected trauma/stress-related genes revealed the differential iper-methylation of brain-derived neurotrophic factor, dopamine receptor D2 and insulin-like growth factor 2 genes. These genes are among those related to brain plasticity, learning, and memory pathways. Conclusions: The association of early detection of posttraumatic distress and epigenetic marker identification could represent a new avenue for addressing women survivors toward resilience. This innovative approach in gender-based violence studies could identify new molecular pathways associated with the long-term effects of violence and implement innovative protocols of precision medicine.

## 1. Introduction

Violence against women is a significant health and social problem whose psychological consequences may have destructive consequences on women’s life. Violence against women runs across all social classes and ethnicity, and it is associated with a considerable negative influence on women’s health and behavior worldwide. The United Nations defines violence against women as “any act gender-based violence that results in or is likely to result in, physical, sexual, or mental harm or suffering to women, including threats to such acts, coercion or arbitrary deprivation of liberty, whether occurring in public or in private life” [1]. A recent observational study referred that about 51.7% of women in the European Union have been victims of violence in their lifetime. The prevalence of physical, sexual, and psychological IPV was, respectively, 20.0% (19.6–20.4), 8.4% (8.2–8.7), and 48.5% (48.1–49.0) [2], with long-lasting health adverse consequences [3,4,5].

Among all forms of violence against women, intimate partner violence (IPV) is the most prevalent. IPV has been defined, by the Centers for Disease Control and Prevention (CDC), as violence that includes physical violence, sexual violence, stalking, and psychological aggression perpetrated by a current or former intimate partner [6]. IPV survivors suffer various consequences that range from higher morbidity and mortality and physical and psychological health problems [7]. There is a consistent and growing body of research on the associations between IPV and women’s mental health problems, in particular Post Traumatic Stress Disorder (PTSD), that appears to be influenced by several multiple factors, such as the type, duration, and severity of violence [8]. PTSD is a stress-related psychiatric disorder triggered by sudden traumatic events and multiple genomic factors. PTSD symptoms may include severe anxiety, flashbacks, nightmares, symptoms of increased arousal such as irritability or anger, or symptoms of persistent avoidance of trauma-related situations [9].

### 1.1. Epigenetic Changes and PTSD

Traumatic experiences, such as, for instance, gender violence, seem to potentially affect genome regulation and expression by epigenetic modification as a response to trauma [10]. Epigenetics refers to gene expression modifications that are heritable, do not involve changes in gene function, and are not attributed to alterations of the DNA sequence. Epigenetic changes include DNA methylation, modifications of histone proteins, and small RNA-mediated gene silencing (miRNAs), which affect gene expression. The remarkable growth in understanding epigenetic mechanisms and the impact of epigenetics on contemporary biology has added new insight into the molecular processes that connect the brain with behavior, neuroendocrine responsiveness, and immune outcome [11]. In this context, the relationship between PTSD and the presence of epigenetic marks in different genes was highlighted. Some of the genes are involved in the regulation of the hypothalamic-pituitary-adrenal (HPA) axis [12,13].

Differentially regulated methylation levels of genes associated with the hypothalamic-pituitary-adrenal (HPA) axis, neurotransmission, neuroplasticity, and inflammation genes were linked to PTSD [14,15,16,17,18,19]. Both early detection of IPV and the significant number of PTSD cases represent a priority area of research, which is focused not only on susceptibility but also on resilience to PTSD. Knowing the mechanisms responsible for resilience, we can derive clues about the best treatment and the best public policy to adopt. Initial evidence on the associations between the presence of epigenetic marks (such as DNA methylation at CpG dinucleotide sites) and PTSD after trauma exposure is available, suggesting a role for epigenetic regulation of physiological and behavioral responses to environmental stressors [16,20]. Noteworthy, epigenetic modifications are potentially reversible, and this specific feature makes epigenetic effectors an attractive target for treatments.

The correlation between PTSD and epigenetic modifications is now being studied concerning various categories of subjects, such as war veterans, abused children, and holocaust survivors [21,22]. Studies on women war veterans have widely demonstrated that violence can impact health by inducing molecular modifications at the epigenetic level, contributing to the onset of mental, physical, and chronic diseases [23,24]. Few studies have examined the relationship between IPV, PTSD, and epigenetic changes in non-veteran women; thus, longitudinal studies or systematic reviews for any health associations with IPV and epigenetic modifications are not present in the literature. Practically, epigenetics gives a long-term picture of the effects of the environment on disease risks, providing significant leverages for designing strategies for the health of all populations [25].

PTSD shows a clear genetic component, as demonstrated by twin studies that report a 24 to 72% heritability following trauma exposure, suggesting that PTSD is a complex multifactorial pathology with an inherent polygenic predisposition. However, genetic aspects of PTSD pathogenesis are not fully characterized, and implicated genes are mainly identified by Genome-Wide Association Studies (GWAS) [26].

### 1.2. Aim of the Study

This study is a pilot study called EpiWe (Epigenetics for WomEn), aimed at seeking epigenetic signatures related to PTSD associated with violence against women. This pilot study represents a preliminary attempt to link stress-related disorders in women who have been exposed to IPV or sexual violence to epigenetic changes observed in their blood samples.

First, we evaluated PTSD symptoms in a sample of women victims of IPV and sexual violence, assisted in a public against violence center during the year 2019. In a second phase, methylation analysis of a panel of ten trauma/stress-related genes was performed on a sample of women aged 18–65 years who filled out proper informed consent and completed detailed questionnaires about PTSD and other health-related data. The objective was to investigate possible IPV-operated epigenetic modulation of a set of genes selected from those highlighted by GWAS to strengthen their role in PTSD pathogenesis. The initial project involved the collection of blood samples for epigenetic analysis at study entry (time 0), at 6, 12, and 18 months after the first evaluation.

The epigenetics of violence against women represents an innovative effort to implement the existing multidisciplinary actions required for reducing this chronic and global health plague with the optimization and individualization of therapies and, from a forward-looking perspective, allow better evaluation of bodily damages in medico-legal settings.

## 2. Materials and Methods

The study was conducted at SVSeD (Service for Sexual and Domestic Violence). SVSeD is a public against violence center located in a university hospital, Department of Women’s and Children’s Health, Fondazione IRCCS Ca’ Granda, Ospedale Maggiore Policlinico, Milan, Italy. SVSeD offers health, social, psychological, forensic, and legal support to women and children exposed to sexual abuse and IPV. All different professional figures cooperate in a well-established, standardized, and comprehensive multidisciplinary approach. All women, in case of sexual abuse or physical injuries, are offered a gynecological and/or clinical/forensic examination during their first access. SVSeD offers immediate psychological support, especially to women suffering from psychological violence. Moreover, SVSeD offers free legal assistance to victims of violence who want to report their cases to the police. Social workers and doctors working at SVSeD usually directly refer to the judicial authority in the cases specified by Italian law.

Institutional Review Board approval for this study was obtained (Ethical Committee of the Italian National Institute of Health (Prot 23/07/2015-0022568) and the Ethical Committee of Fondazione IRCCS Ca Granda Ospedale Maggiore Policlinico (Prot 18/05/2016—258_2016 bis, Milan, Italy).

All women participating in the current research project signed a specific written informed consent before enrolment. In addition, all women accessing for the first time to SVSeD are routinely requested to sign written informed consent to clinical/forensic procedures, as well as to authorize researchers to review their medical records and use their data for research, ensuring data confidentiality.

### 2.1. PTSD Prevalence Study

We conducted an exploratory retrospective study to estimate the PTSD prevalence in a sample of 914 victims of sexual violence and IPV who were admitted to SVSeD in 2019.

In SVSeD usual clinical practice, the evaluation of the psychological status of the survivors of gender violence, including PTSD, is always performed during the first clinical interview and reported in the clinical record.

For this research, data regarding intrusive symptoms of PTSD (such as recurrent, unwanted, and distressing thoughts of the traumatic event), avoidance symptoms (i.e., efforts to avoid feelings, activities, places, or people that increase reminders of the trauma), and symptoms of hyperarousal/physiological activation, such as sleep difficulty, irritability, hypervigilance, difficulty in concentration, were retrospectively collected by analyzing women’s SVSeD medical records.

Sexual violence is often a single event, occurring suddenly, while IPV is, by definition, a long-lasting sequence of events. Following DSM-V criteria, PTSD symptoms must last for more than a month, so the evaluation of victims of sexual violence has been postponed (at least one month) after the acute traumatic event to meet DSM-V criteria.

### 2.2. Epigenetic Study

#### 2.2.1. Questionnaire

During the year 2020 only, we conducted an epigenetic study (blood sampling and specific questionnaires) including a group of 62 women exposed to violence (13 women suffering from sexual violence and 49 from IPV) and 50 women with no history of violence. This group of women was different from the one involved in the PTSD prevalence study. The group of women survivors of sexual violence/IPV enrolled in the epigenetic study was aged 18–65. The non-exposed group comprises women (aged 18–65) with no history of violence or severe pathologies and/or trauma.

Trauma-exposed women were slightly younger than non-exposed ones, with an average age of 37 and 43, respectively. The questionnaire encompasses the following main questions: type and severity of violence, risk of recurrence (SARA—SV screening), sociodemographic data, social and relational condition, anamnestic data with drug abuse, self-harm risk, PCL-C PTSD checklist (6 items), revised CESD-R (10 items), medical outcome short form survey 12.

We conducted descriptive univariate analyses of selected information by the questionnaire, including sociodemographic characteristics and other factors that could modify the epigenetic profile overall and in the group of trauma-exposed and non-exposed women separately. When appropriate, categorical variables were presented as frequencies and proportions, while continuous variables were represented as mean and standard deviation or median and interquartile range. Differences in baseline characteristics between the groups were tested using parametric tests for normal distributions (e.g., Chi-squared test or Students *t*-test) or non-parametric tests for skewed distributions (e.g., McNemar’s test or Wilcoxon rank-sum test).

The physical and psychological conditions of the women were assessed (health conditions, PTSD, and depression), the PTSD score was assessed with the PCL-C scale (abbreviated PTSD Checklist for Civilians), and values equal to or greater than 14 were considered to determine the PTSD prevalence. The difference in PTSD prevalence between the groups was tested using the Chi-squared test.

#### 2.2.2. Blood Collection (DNA Samples)

The blood sample was collected in a 6 mL EDTA plasma tube, and genomic DNA extraction was performed using the DNeasy DNA blood kit (Qiagen, Hilden, Germany). The collection of blood was performed 6–7 days after sexual violence took place, while in the case of IPV, the collection of blood samples was performed the same day of hospital admission.

#### 2.2.3. DNA Methylation Analysis

The genetic architecture that underlines PTSD is complex and involves many genes, probably not yet all identified. We chose a panel of loci already characterized in the stress disorder and PTSD cohort studies and reported as relevant in psychiatric conditions and posttraumatic stress [14,27,28,29,30,31,32]: *ADCYAP1*, *BDNF*, *CRHR1*, *DRD2*, *FKBP5*, *IGF2*, *LSD1*, *NR3C1*, *PRTFDC1*, *SLC6A4.* Promoter regions were selected based on previously published data and in silico prediction of promoter sequences [33]. PCR primers have been designed for each promoter using the EpiDesigner software [34] to amplify multiple CpGs (Table 1). The quantitative analysis of DNA methylation will be carried out with the MassARRAYAnalyzer 4_MALDI_TOF mass spectrometer (Agena Bioscience) and the EpiTYPER software. Results are provided with values ranging from 0 to 1, indicating unmethylated or methylated CpGs, respectively. Table XYZ indicates the genomic position of PCR primers, the amplicon size, the number of CpG sites within the PCR product, and the CpG coverage, meaning the number of CpG sites quantitatively analyzed by the software.

#### 2.2.4. Statistical Analysis

Differences in DNA methylation between groups were tested using non-parametric tests (i.e., Wilcoxon rank-sum test) since the variables were non-normally distributed.

## 3. Results

### 3.1. PTSD Prevalence Study

In 2019, 914 victims of violence were admitted to the SVSeD. All women at the first access to SVSeD were offered a psychological interview, with a narrative synthesis reported in the medical record. Specifically, data on PTSD were available only regarding 496 of 914 (54.2%) women who completed the psychological follow-up and the PTSD form.

Of 496 women, 40 (8.1%) had a diagnosis of PTSD, 16 of those were victims of sexual violence, and 24 were victims of IPV. In addition, 73 (14.7% of 496) patients presented at the visit with at least 1 or more diagnostic criteria associated with PTSD, 33 of those were victims of sexual violence, and 40 were victims of IPV. Among women with PTSD, 40% have experienced sexual violence, and 60% with IPV. When considering women with at least one PTSD diagnostic criterion, the percentage was 45.2% and 54.8%, respectively (Table 2).

### 3.2. Epigenetic Study

Quantitative methylation evaluation of a panel of selected genes associated with trauma/stress-related disorders, including *ADCYAP1*, *BDNF*, *CRHR1*, *DRD2*, *FKBP*, *IGF2*, *LSD1*, *NR3C1*, *PRTFDC1*, *SLC6A4*, was performed.

Differences in DNA methylations between trauma-exposed women and women free of trauma emerged for the BDNF, DRD2, and IGF2 genes (Table 3). When we considered the subgroup of women exposed to IPV (*n* = 49), the BDNF and IGF2 genes remained significantly different (data not shown).

## 4. Discussion

The present research was performed starting by observing the results described in PTSD epigenetic studies related to combat veterans of war and natural disaster survivors. The hypothesis of this pilot study is that violence against women, like major traumas, can be considered a powerful trigger of PTSD.

First, we retrospectively evaluated the PTSD prevalence in the SVSeD one-year sample of victims of sexual violence and IPV, and we found a prevalence of PTSD of about 8.1%. Second, we compared the epigenetic signatures for selected genes between the exposed victims and the non-exposed ones (control group) to identify epigenetic signatures of violence-related trauma. The final goal was to search for a more robust aid in predicting the onset of the severity of the psychological consequences of violence. Comparing the epigenetic differences between victims and controls, we have shown significant changes in the promoter methylation of *BDNF*, *DRD2*, and *IGF2* genes.

*BDNF* gene encodes for the brain-derived neurotrophic factor, the major synaptic transmission and neuroplasticity regulator. It is involved in stress response, learning, and memory [35]. Studies on rats evidenced that exercise induces hypomethylation in the *Bdnf* promoter, thus increasing its expression [36]. This evidence suggests that it could represent a potential actionable target. Our results confirm that hypermethylation of this locus is associated with stress since analogous results were found in veterans with PTSD [37].

*DRD2* findings are also in accordance with other observations. Groleau et al. [38] reported that women with a bulimia-spectrum disorder with a history of childhood sexual abuse showed a trend-level elevation of *DRD2* methylation, which could lead to lower dopaminergic functioning at the D2 receptors. A finding suggests an association with persistent epigenetic modification at this locus after sexual abuse.

The third locus significantly hypermethylated was *IGF2*, a gene regulated by genomic imprinting which encodes for insulin factor 2, considered the master gene of fetal and placental growth [39]. Besides, IGF2 also has important effects on multiple brain functions: memory, depression, and autism. Several studies have shown in an animal model that IGF2 treatment enhances memory functions and reverses cognitive impairment, motor deficits, and seizures in affected mice [40,41]. In addition, Mellott et al. [42] showed that intracerebral injection of the protein in mice up-regulated levels of BDNF in the hippocampus, thus suggesting a common pathway between the two genes showing significant epigenetic modifications in cases compared to control women. In addition, the STRING database [43] also reports a co-expression of hippocampal BDNF and DRD2 protein.

These findings suggest that BDNF expression is crucial in response to IGF2 stimulus and that there is a functional link between BDNF and DRD2. These findings, although preliminary, are promising in revealing epigenetic markers in genes mediators of brain plasticity, which can modulate learning and memory in response to stress associated with intimate partner and sexual violence-induced PTSD.

### Limitation of the Study

The limited number of victims and control cases analyzed has prevented us from evaluating a statistically significant association between the clinical status of the victims and their epigenetic signature type. Moreover, the PTSD prevalence study was conducted retrospectively, and this study design carries the typical limitations of recorded patient information, including a high rate of unreported information and possible errors.

## 5. Conclusions

Starting from the initial evidence that emerged from the present pilot study, our EpiWE project will be proposed as a multicenter study to enroll a significant number of victims to search for correlations between PTSD symptoms and epigenetic signatures. We will investigate epigenetic signatures by epigenome-wide approaches to increase knowledge in this research’s field and comprehensively investigate the role of epigenetic modifications in patients with stress-related disorders, particularly PTSD.

By this approach, we aim to identify epigenetic signatures of violence that can be translated into clinical practice, aiming to better predict the severity of the stress-related psychological consequences of gender violence.

The differentially expressed genes could also play a role in other biological pathways related to the long-term health effects of violence (e.g., cancer, cardiovascular and autoimmune diseases). By contributing to the knowledge of molecular mechanisms underlying PTSD in the context of violence against women, we could derive clues about better treatments and better public health policies.

## Figures and Tables

**Table 1:** ^1^ chromosome location, ^2^ reference sequences, ^3^ genomic positions, ^4^ CpG sites within the investigated region, ^5^ CpG sites quantified by the EpiTYPER software, and ^6^ amplicon lengths of the investigated loci.

Gene	Chromosome ^1^	Sequence ID ^2^	Start–End ^3^	N° CpG Sites ^4^	CpG Coverage ^5^	PCR Product Size (bps) ^6^
*ADCYAP1*	18	AP000894.6	110,108–110,333	10	9	226
*BDNF*	11	NG_011794.1	4002–4350	22	21	349
*CRHR1*	17	NG_009902.1	3971–4263	24	21	291
*DRD2*	11	NG_008841.1	4581–4789	12	12	209
*FKBP5*	6	NG_012645.2	35,687,781–35,688,046	15	10	266
*IGF2*	11	NG_008849.1	21,346–21,832	29	25	487
*LSD1/KDM1A*	1	NG_047129.1	5217–5464	25	18	248
*NR3C1*	5	NG_009062.1	36,173–36,575	47	27	403
*PRTFDC1*	10	NC_000010.11	24,953,017–24,953,295	6	4	279
*SLC6A4*	17	NG_011747.2	4906–5202	29	20	297

**Table 2:** Distribution of type of violence in the sample of women with PTSD (*n* = 40) and those who had at least one diagnostic criterion of PTSD (*n* = 73).

	Women with a Diagnosis of PTSD N = 40	Women Who Had at Least One Diagnostic Criterion N = 73	Total N = 113
	N (%)	N (%)	N (%)
Type of violence			
Sexual	16 (40.0)	33 (45.2)	39 (34.5)
IPV	24 (60.0)	40 (54.8)	64 (56.6)

**Table 3:** Distribution (median and interquartile range) of DNA methylation of 10 genes among 62 trauma-exposed women and 50 trauma-free controls.

	Exposed (*n* = 62)			Non Exposed (*n* = 50)			
	I Quartile	Median	III Quartile	I Quartile	Median	III Quartile	*p*-Value *
*ADCYAP1*	0.11	0.13	0.15	0.11	0.13	0.16	0.307
*BDNF*	0.07	0.08	0.10	0.06	0.07	0.08	0.002
*CRHR1*	0.07	0.08	0.08	0.07	0.08	0.08	0.496
*DRD2*	0.10	0.14	0.17	0.08	0.11	0.14	0.003
*FKBP5*	0.04	0.05	0.05	0.05	0.05	0.06	0.018
*IGF2*	0.49	0.53	0.56	0.45	0.48	0.52	0.001
*LSD1*	0.04	0.04	0.05	0.03	0.04	0.06	0.186
*NR3C1*	0.03	0.04	0.05	0.03	0.04	0.05	0.188
*PRTFDC1*	0.03	0.04	0.05	0.04	0.04	0.05	0.271
*SLC6A4*	0.04	0.05	0.05	0.04	0.05	0.05	0.347

* Non-parametric Wilcoxon rank-sum test (since Bonferroni’s correction for multiple comparisons is adopted, a *p*-value < 0.005 (0.05/10) indicates that the median is statistically different between groups). BDNF, DRD2, and IGF2 resulted in hypermethylated in trauma-exposed women.

## Data Availability

Not applicable.
